# Description of *Prevotella rectalis* sp. nov., a new bacterium isolated from human rectum

**DOI:** 10.1016/j.nmni.2020.100703

**Published:** 2020-05-28

**Authors:** S. Belkacemi, C.I. Lo, S. Khelaifia, D. Raoult, P.-E. Fournier, F. Fenollar

**Affiliations:** 1) Aix Marseille Université, IRD, AP-HM, MEPHI, Marseille, France; 2) IHU-Méditerranée Infection, Marseille, France; 3) Aix Marseille Université, IRD, AP-HM, SSA, VITROME, Marseille, France

**Keywords:** Culturomics, Human gut, *Prevotella rectalis*sp. nov., Rectum

## Abstract

Using a taxonogenomics method, we describe here a Gram-negative bacterium named *Prevotella rectalis* sp. nov., strain Marseille-P4334^T^ (= CSUR P4334) isolated from the rectum. Strain Marseille-P4334^T^ has a genome that measure 3.03 Mbp with 43.3 mol% G + C content.

## Introduction

Studying the diversity of bacteria is fundamental to understanding their involvement in human diseases and normal physiological functions [1]. To unveil human microbial diversity, the culturomics approach, based on diversified culture conditions, was designed to isolate as yet uncultured species and to complement 16S rRNA metagenomics [[2], [3], [4]]. Furthermore, a new taxonomic strategy, named taxonogenomics, was developed to include the analysis of complete genome sequences in combination with phenotypic characteristics [5]. Herein, we report a detailed description of *Prevotella rectalis* sp. nov., for which the type strain Marseille-P4334^T^ was isolated from the human rectum.

### Isolation and growth conditions

**Fig. 1:**
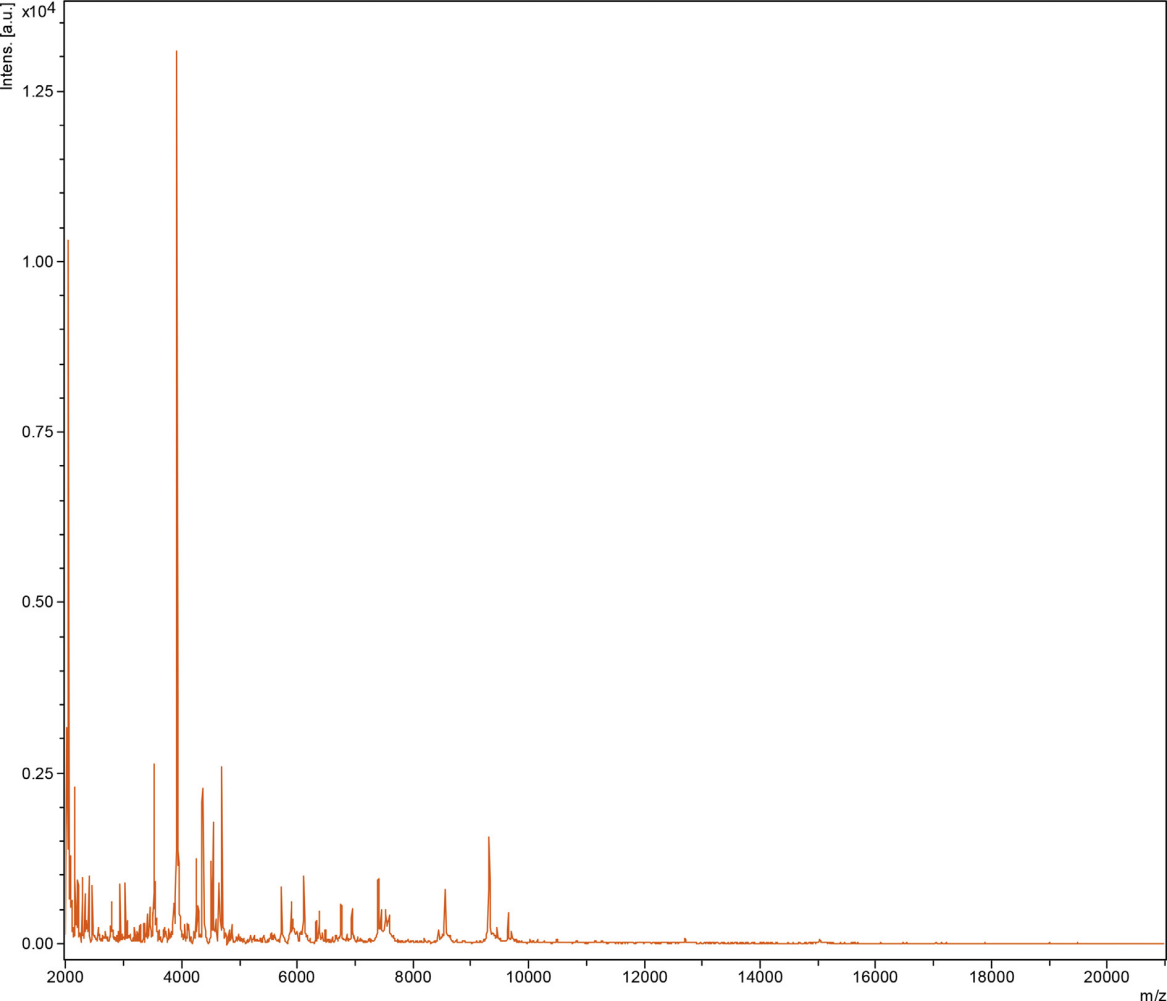
MALDI-TOF MS reference spectra of *Prevotella rectalis* sp. nov., strain Marseille-P4334^T^. Separately the two reference spectra were generated by comparison of spectra from 12 individual colonies from each strain.

In 2017, we isolated an unidentified bacterium strain from a human rectum. The study was approved by the Institut Federatif de Recherche 48 (agreement number 09-022, Marseille, France), and the patient's consent was obtained. After three tests using matrix-assisted laser desorption/ionization time of flight mass spectrometry (MALDI-TOF MS), the strain was not identified. Screening was carried out on a Microflex LT spectrometer (Bruker Daltonics, Bremen, Germany) as previously described [6]. Spectra obtained from the strain (Fig. 1) were imported and analysed using Biotyper 3.0 software against the Bruker database that was permanently updated with the local MEPHI database (https://www.mediterranee-infection.com/urms-data-base).

The growth of strain Marseille-P4334^T^ was possible on 5% sheep's blood-agar (bioMérieux, Marcy l’Étoile, France) under anaerobic atmosphere (anaeroGEN; Oxoid, Dardilly, France) at 37°C after 3 days of pre-incubation and 5 days in incubation.

### Strain identification

**Fig. 2:**
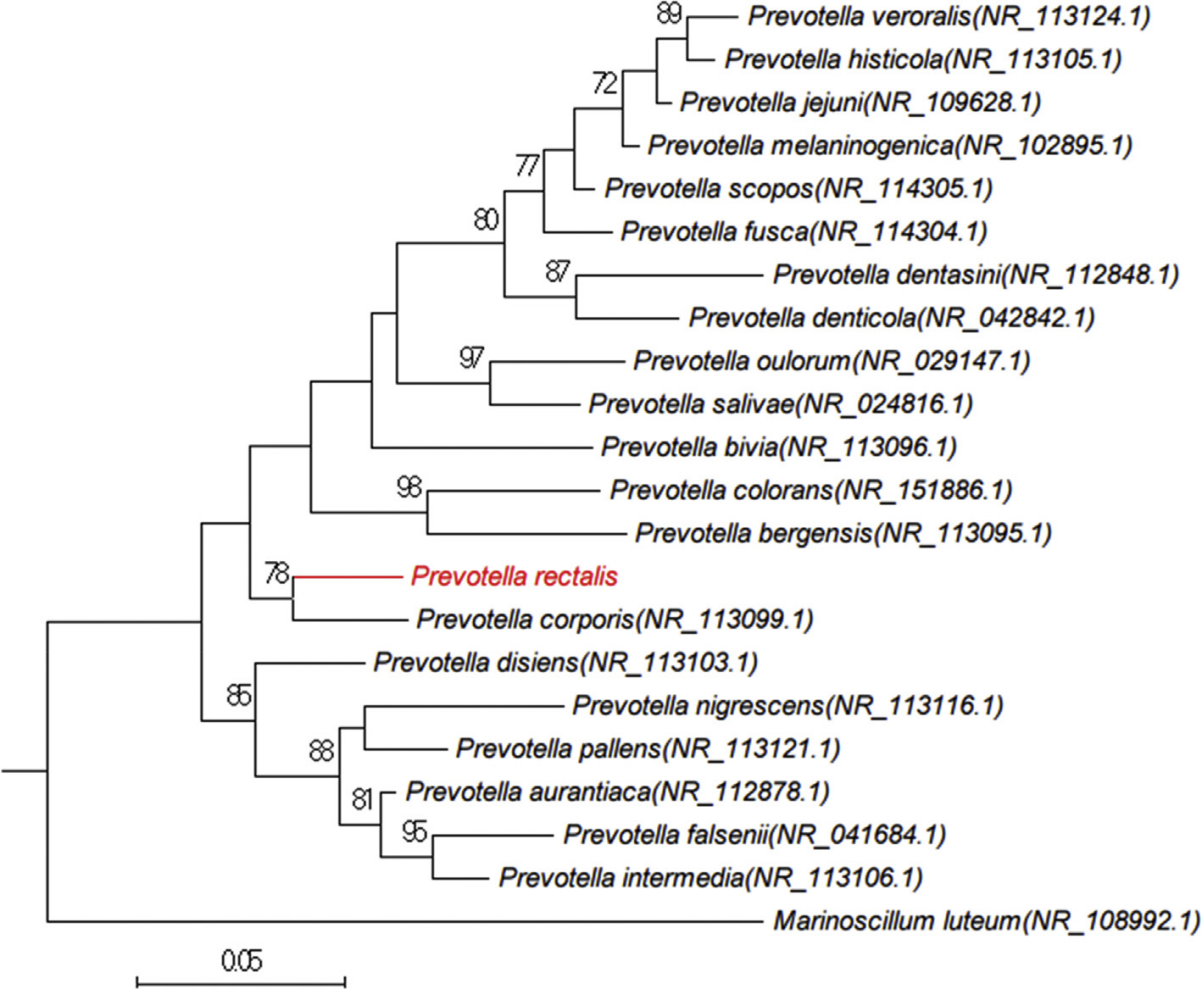
Phylogenetic tree highlighting the position of *Prevotella rectalis* sp. nov. strain Marseille-P4334^T^ with regard to other closely related species. GenBank accession numbers of 16S rRNA are indicated in parentheses. Sequences were aligned using MUSCLE with default parameters, phylogenetic inferences were obtained using the maximum likelihood method and MEGA 7 software. Bootstrap values obtained by repeating the analysis 1000 times to generate a majority consensus tree are indicated at the nodes.

The 16S rRNA gene was sequenced to classify this bacterium. Amplification was performed using the primer pair fD1 and rP2 (Eurogentec, Angers, France) and sequenced using the Big Dye® Terminator v1.1 Cycle Sequencing Kit and 3500xLGenetic Analyzer capillary sequencer (Thermofisher, Saint-Aubin, France), as previously described [7]. The 16S rRNA nucleotide sequence was assembled and corrected using CodonCode Aligner software (http://www.codoncode.com). Strain Marseille-P4334^T^ exhibited a 96.4% rRNA sequence identity with *Prevotella corporis* strain JCM 8529 (GenBank accession number: NR_113099.1), its phylogenetically closest species with standing in nomenclature (Fig. 2). Considering this value less than a threshold value established previously [8], we consequently proposed to classify strain Marseille-P4334^T^ as a new species within the genus *Prevotella* in the phylum *Bacteroidetes.*

### Phenotypic characteristics

**Fig. 3:**
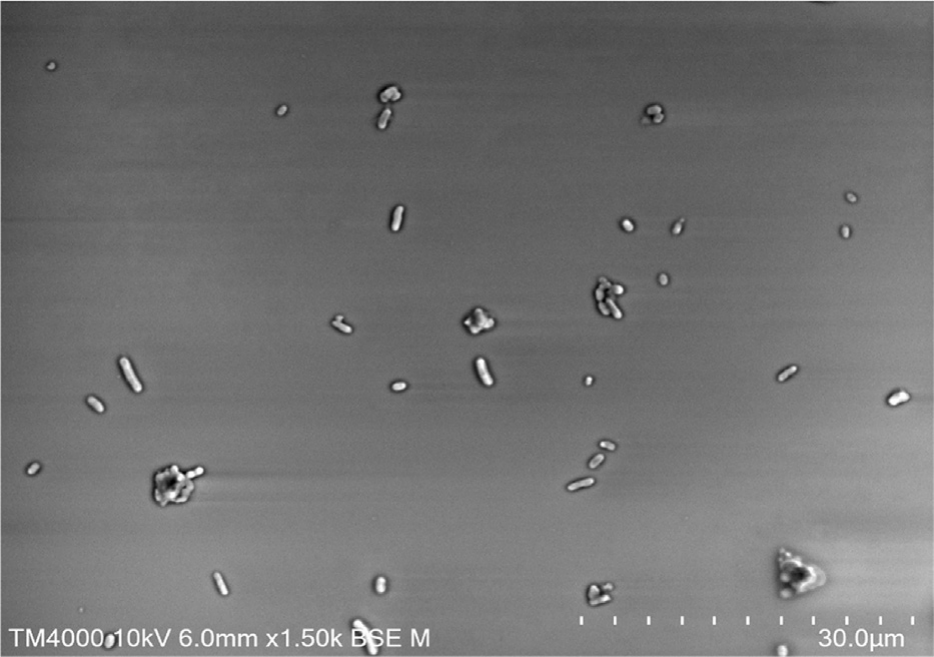
Scanning electron microscopy (SEM) of the new species. For each strain a colony was collected from agar and immersed into a 2.5% glutaraldehyde fixative solution. Then, a drop of the suspension was directly deposited on a poly-l-lysine-coated microscope slide for 5 minutes and treated with 1% phosphotungstic acid aqueous solution (pH 2.0) for 2 minutes to increase the SEM image contrast. The slide was gently washed in water, air-dried and examined in a tabletop SEM (Hitachi TM4000). Scales and acquisition settings are shown on the figures.

Colonies of strain Marseille-P4334^T^ were black and smooth with a mean diameter of 0.5–1 mm. Bacterial cells were Gram-negative bacilli ranging from 1 to 3 μm in length and 0.4 to 0.6 μm in width. Strain Marseille-P4334^T^ showed catalase-positive and oxidase-negative activities. Electron micrograph showing the shapes of this new bacterial strain (Fig. 3) was performed with the Hitachi TM4000 instrument (Hitachi Group, Krefeld, Germany). A comparative study of the biochemical characteristics of strain Marseille-P4334^T^ with other closely related species in phylum *Bacteroidetes* is presented in Table 1. Results of biochemical tests performed using the API ZYM and 50 CH strips (bioMérieux) are shown in Table 2.

**Table 1:** Differential characteristics of *Prevotella rectalis* sp. nov., strain Marseille-P4334^T^ compared with related species within their respective genus

Properties	*Prevotella rectalis*	*Prevotella multiformis*	*Prevotella bivia*	*Prevotella melaninogenica*	*Prevotella aurantiaca*
Gram stain	—	—	—	—	—
Motility	—	—	—	—	—
Endospore formation	—	—	—	—	—
Catalase	+	—	NA	NA	—
Glycerol	—	+	—	—	—
d-Cellobiose	—	+	—	—	—
d-Raffinose	—	+	—	+	+
α-Galactosidase	+	—	—	NA	—
β-Galactosidase	—	+	+	+	—
α-Fucosidase	—	NA	+	+	+
α-Glucosidase	—	—	NA	+	NA
β-Glucosidase	+	+	+	+	NA
Arginine aminopeptidase	—	—	—	+	NA
Source	Human rectal	Subgingival plaque	Vagina	Human respiratory tract	Human oral cavity

+, positive result; −, negative result; NA, data not available.

**Table 2:** Phenotypic characterizations of *Prevotella rectalis* sp. nov., based on API tests

API tests	Characteristics	P4334^T^
ZYM	Alkaline phosphatase	—
	Esterase (C4)	+
	Esterase lipase (C8)	+
	Lipase (C14)	—
	Leucine arylamidase	—
	Valine arylamidase	+
	Cystine arylamidase	—
	Trypsin	—
	α-Chymotrypsin	—
	Acid phosphatase	—
	Naphthol-AS-BI-phosphohydrolase	+
	α-Galactosidase	+
	β-Galactosidase	—
	β-Glucuronidase	—
	α-Glucosidase	—
	β-Glucosidase	+
	*N*-Acetyl-β-glucosaminidase	—
	α-Mannosidase	—
	α-Fucosidase	—
	2-Nitrophenyl βd-galactopyranoside	—
50 CH	Glycerol	—
	Erythritol	—
	d-Arabinose	—
	l-Arabinose	—
	d-Ribose	—
	d-Xylose	—
	l-Xylose	—
	d-Adonitol	—
	Methyl βd-Xylopyranoside	—
	d-Galactose	+
	d-Glucose	—
	d-Fructose	—
	d-Mannose	—
	l-Sorbose	—
	l-Rhamnose	—
	Dulcitol	—
	Inositol	—
	d-Mannitol	—
	d-Sorbitol	—
	Methyl-αd-Mannopyranoside	—
	Methyl-αd-Glucopyranoside	—
	*N*-Acetylglucosamine	—
	Amygdalin	—
	Arbutin	—
	Esculin ferric citrate	—
	Salicin	—
	d-Cellobiose	—
	d-Maltose	—
	d-Lactose	—
	d-Melibiose	—
	d-Saccharose	—
	d-Trehalose	—
	Inulin	—
	d-Melezitose	—
	d-Raffinose	—
	Amidon	—
	Glycogen	—
	Xylitol	—
	Gentiobiose	—
	d-Turanose	—
	d-Xylose	—
	d-Tagalose	—
	d-Fucose	—
	l-Fucose	—
	d-Arabitol	—
	l-Arabitol	—
	Potassium gluconate	—
	Potassium 2-ketogluconate	—
	Potassium 5-ketogluconate	—

### Genome sequencing

**Fig. 4:**
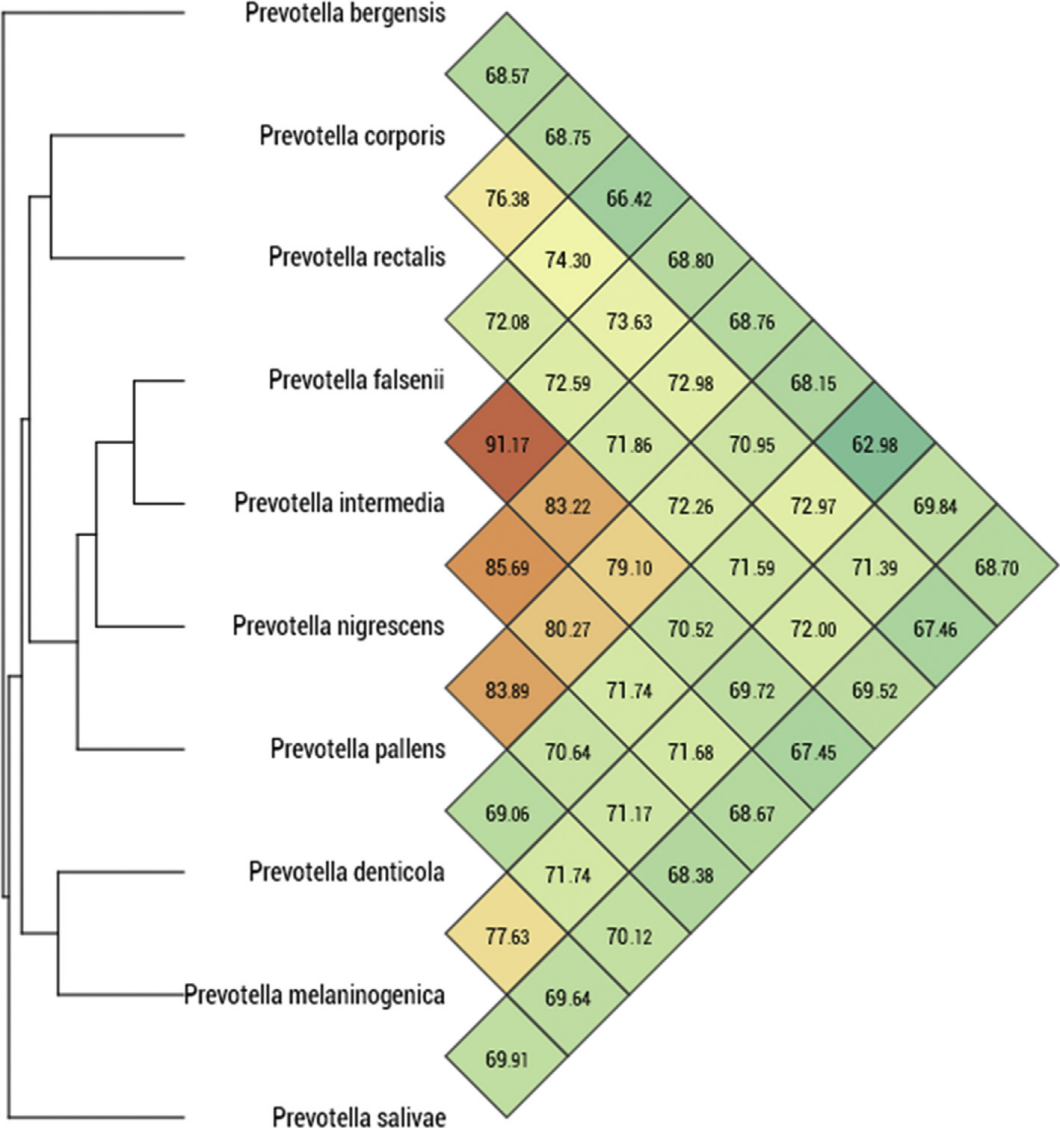
Heatmap generated with OrthoANI values calculated using the OAT software for *Prevotella rectalis* sp. nov., strain Marseille-P4334 compared with other closely related species with standing in nomenclature.

Genomic DNA was extracted using the EZ1 biorobot with the EZ1 DNA tissue kit (Qiagen, Hilden, Germany) and then sequenced on a MiSeq sequencer (Illumina Inc., San Diego, CA, USA) with the Nextera Mate Pair sample prep kit and Nextera XT Paired End (Illumina), as previously described [9]. The assembly was performed using a pipeline containing several softwares (Velvet [10], Spades [11] and Soap Denovo [12]), and trimmed data (MiSeq and Trimmomatic [13] softwares) or untrimmed data (only MiSeq software). GapCloser was used to reduce assembly gaps. Scaffolds <800 bp and scaffolds with a depth value < 25% of the mean depth were removed. The best assembly was selected using different criteria (number of scaffolds, N50, number of N). Strain Marseille-P4334^T^ had a genome size of 3 039 397 bp with 43.3 mol% G + C content. The degree of genomic identity of this strain with closely related species was calculated using OrthoANI software [14]. Results of this analysis are displayed in Fig. 4. For strain Marseille-P4334^T^, OrthoANI values among closely related species ranged from 68.57% between *Prevotella corporis* and *Prevotella bergensis* to 68.70% between *Prevotella bergensis* and *Prevotella salivae*. When *P. rectalis* was compared with these closely related species, values ranged from 69.91% with *P. salivae* to 76.38% with *P. corporis*.

## Conclusion

On the basis of unique phenotypic features, including the MALDI-TOF spectrum, a 16S rRNA sequence divergence >1.3% and an OrthoANI value > 95% with the phylogenetically closest species with standing in nomenclature, we formally proposed strain Marseille-P4334^T^ as the type strain of *Prevotella rectalis* sp. nov., as a new member within the phylum *Bacteriodetes.*

## Description of *Prevotella rectalis* sp. nov

*Prevotella rectalis* (rec.ta'lis. L. gen. n. *rectalis* pertaining to rectum, the chamber that begins at the end of the large intestine from which this bacterium was isolated). It is a Gram-negative bacterium, non-motile and non-spore-forming. Strain Marseille-P4334^T^ is the type strain of *Prevotella rectalis* sp. nov., and shows catalase-positive and oxidase-negative activities. The strain grows strictly under anaerobic conditions at 37°C. The potential pathogenicity of the type strain Marseille-P4334^T^ (= CSUR P4334) is unknown. This strain has a genome size of 3.03 Mbp long with a 43.3 mol% G + C content. The 16S rRNA gene sequence and whole-genome shotgun sequence of strain Marseille-P4334 were deposited in GenBank under accession numbers LS488976 and UWTY00000000, respectively. It has been isolated from the human rectum.

## Conflicts of interest

None to declare.

## Funding sources

This study was supported by the Institut Hospitalo-Universitaire (IHU) 10.13039/100007356Méditerranée Infection, the 10.13039/501100001665National Research Agency under the programme *Investissements d'avenir*, reference ANR-10-IAHU-03, the 10.13039/501100010076Région Provence-Alpes-Côte d'Azur and European funding FEDER PRIMI.
